# Bibliographical Notices

**Published:** 1846-05

**Authors:** 


### BIBLIOGRAPHICAL NOTICES.

*Clinical Lectures on Surgery, delivered at St. George's Hospital.* By Sir Benjamin Brodie, Bart, V. P. R. S., Sergeant Surgeon to the Queen, etc. etc. 8vo. pp. 352. Lea & Blanchard. Philadelphia: 1846.

*Lectures on the Nature and Treatment of Deformities, Delivered at the Royal Orthopœdic Hospital, Bloomsbury Square.* By R. W. Tamplin, F. R. C. S. E., Surgeon to the Hospital. *With numerous illustrations.* 8vo. pp. 216. Ed. Barrington & George D. Haswell. Philadelphia: 1846.

*Elements of Surgery.* By Robert Liston, Surgeon to the North London Hospital, Professor of Clinical Surgery, etc. etc. *Fourth American from the last London Edition, with copious notes and additions.* By Samuel D. Gross, M. D., Professor of

Surgery in the Medical Institute of Louisville, Surgeon to the Louisville Marine Hospital, etc. etc. *Illustrated with numerous engravings.* 8vo. pp. 664. Edward Barrington & George D. Haswell. Philadelphia: 1846.

*Lectures on the Operations of Surgery, and on Diseases and Accidents requiring Operations.* By ROBERT LISTON, Esq., F. R. S., Senior Surgeon to the University College Hospital, and Professor of Clinical Surgery in the College. *With numerous additions,* by THOMAS D. MÜTTER, M. D., Professor of Surgery in Jefferson Medical College of Philadelphia; Fellow of the Royal Medical and Chirurgical Society of London, etc. etc. 8vo. pp. 565. Lea & Blanchard. Philadelphia: 1846.

A venerable friend, and distinguished member of the medical profession, was wont to say that extraordinary cases seldom occur singly—that with accidents and severe surgical cases, the advent of one is but the prelude to others.

Something of the sort not unfrequently happens with medical book-publishing; at one time we have pouring forth volume upon volume of *Practice;* next a flood of *Midwifery, Diseases of Women, and Diseases of Children;* then we have tomes, both great and small, on *Dermatology;* anon we have *Chemistry,* or, it may be, *Materia Medica;* now, as our readers will perceive, is the season for *Surgery.* Of the recent publications on this subject, above we have given the titles of some which we shall now notice briefly, and shall endeavour to do at least equal justice to others on hand, in a future number.

1. First in the order of our enumeration is the work of Sir Benjamin Brodie. Some of the lectures contained in the present volume were presented to our readers in the pages of the Examiner of a former year, from which they are enabled to form for themselves some estimate of the character of the work. Sir Benjamin Brodie has long been distinguished as a surgeon, even among those in the front rank, and may now be regarded as occupying the very first place in Great Britain, if not in the world. It is not as a mere operator that he is distinguished—that, it must be conceded by all right thinking minds, is but a very humble part of the qualifications of a surgeon—but as a profound pathologist and therapeutist. We may be prejudiced or mistaken, but in these higher and really intellectual qualifications, we know of no living surgeon that approaches him. His well considered opinions and practical instructions are conveyed, too, in language so appropriate, so simple, clear, and chaste, that one scarcely knows whether most to admire the excellence of the matter or the beauty of the style in which it is clothed. Theory and practice go hand and hand throughout. Rarely is a precept given without being illustrated by some apposite case, selected from his vast experience, and always in the fewest and most expressive words. Nothing more than is necessary to enforce the point is said; and nothing that is necessary is left untold.

The lectures which constitute the present volume were originally published in some of the London Medical Journals, and subsequently republished in the library department of the Medical News, and now are presented to us in the more connected and agreeable form of a separate volume.

We shall not pretend to criticise the doctrines of so eminent a master as Sir Benjamin Brodie, but content ourselves with giving a few occasional extracts on points of great importance, for the edification of our younger brethren, as well as evidences of the sound views, ethical and scientific, of the distinguished lecturer. Lecture 2d, which is devoted to "illustrations of some important circumstances connected with operative surgery," is especially rich in this respect.

"There is no department of the healing art in which there is so much to interest or to excite both our own profession and the public, as there is in operative surgery. In the greater number of cases of disease treated by other means, it is difficult to say how much of the success obtained belongs to the remedies employed, and how much to the natural powers of the patient's constitution. But it is entirely different in those cases that are the subjects of operations. Recourse is had to this mode of treatment only when nature can go no further; and an operation, so far from being the direction of a natural process to a safe result, is, for the most part, an abrupt and rude interference with whatever nature is about. If a cure arise from an operation, it is to be attributed to that, and to that only: and thus it happens that some of the most splendid results obtained in the healing art are those which are claimed by the operating surgeon.

"But an operation, while it may do good, may also be pro ductive of evil. A man has a stone in the bladder; he is suffering torture; he has nothing but a frightful death to which he can look forward. As the least of two evils, he is contented to submit to the operation of lithotomy: and, it may be, that in the brief space of three minutes he is placed in a situation of perfect comfort, and that in forty-eight hours you are able to declare with confidence that his life is perfectly safe. A man may have a disease in the knee-joint, with carious bone and abscesses; he may be worn out by pain, by perspirations, sleepless nights, and other symptoms of hectic fever. You amputate the limb; and even on that very night he may sleep soundly; there may be no more perspirations, and in a week he may be gaining flesh, and present the aspect of health. But then, on the other hand, there are other cases, in which the patient, after lithotomy, may die within forty-eight hours, although he might have lived—in misery, it is true—had he been let alone, a year or longer. So, in the case of amputation for a diseased knee-joint, the patient, instead of recovering, may die in the course of a few days, and very much sooner than he would have done had not an operation been resorted to.

"This double result of operations adds to the interest which this part of surgery possesses, and to the responsibility which is entailed on those who practice it. But what adds still more to the one and to the other, is this—that it is not only great operations, such as lithotomy, and the amputation of the thigh, that are attended with risk. A man died in this hospital from the consequence of a sting of a bee; and another died, in this hospital also, from those of the bite of a leech. A patient died in consequence of a wound, not an inch in length, on the inside of the knee, made for the purpose of dividing the saphena vein. I have known a patient die from erysipelas that followed the simple operation of cupping; and there have been not a few instances of fatal venous inflammation supervening after a common bleeding in the arm. A lady had a small encysted tumour on her head not larger than a pea. A surgeon who was at that time (for what I am speaking of was many years ago) an eminent man in his profession, removed the tumour, but did it imperfectly. The disease returned, and another surgeon, at that time in large practice also, removed it more effectually. The patient died from erysipelas of the scalp. So others have died from the removal of piles, and other apparently trifling operations."

The following is a description of what the lecturer considers to be necessary to constitute "an accomplished operator." How widely he differs from some celebrated French—and perhaps we might add—English and American operators!

"An accomplished operator! That term may be used in various senses; but I will tell you, before I proceed further, in what sense I use it. I apply it, not to him who looks at his watch to see in how short a space of time an operation may be completed; nor to him who, during an operation, is putting himself in the situation of those who are looking on, considering what they will say, and anxious to appear dexterous in their eyes. According to my notions, he only is an accomplished operator who, before he engages in an operation, looks at all the consequences, both good and bad, which may ensue; and earnestly endeavours to lay his plans so that there may be as great a chance as possible of the former being obtained, and of the latter being avoided; and who, while actually engaged in an operation, thinks neither of himself nor of the by-standers, nor allows any question to arise in his mind, except as to what he should do to bring the case ultimately to a safe termination with the least possible distress to the patient."

The following observations on the "loss of blood" we deem of particular importance. Not only are they just, as it regards surgical operations, but they apply with equal force to obstetrical practice, and the extensive employment of bloodletting in the treatment of inflammatory diseases generally. The supervention of inflammation, especially of a diffuse character, after great loss of blood, although a notorious fact, is not enough considered by many practitioners when combating sthenic forms of disease, and hence the erysipelases, peritonites, dropsies, etc., which so frequently follow.

"The patient may recover from the operation, and the wound may be healed, and yet, where there has been a copious hemorrhage, the constitution of a delicate person, more especially of delicate women, may be so much damaged by it, that it may not recover for some years. After an operation I have sometimes heard a bystander say, "oh, he has lost no more blood than it will do him good to lose." It is painful to me to hear such an observation as this: be assured that an operation cannot be performed with too little loss of blood. The loss of a few ounces in a patient who has a stone in the bladder, complicated with disease of the kidneys, will make all the difference between life and death: and so it is in many other cases. If it is desirable that the patient should lose blood, you can always take it from his arm, and just as much as is wanted, and no more. There can at any rate be no advantage from the loss of an uncertain quantity of blood in an operation. Some people seem to me to have a notion that the loss of blood in an operation will make the patient less liable to inflammation afterwards. But I believe that it is just the reverse. Bleeding may relieve phlegmonous inflammation where it already exists, but it does not prevent its existence; and on the other hand, I have no doubt that it increases the liability of the patient to other kinds of inflammation, such as erysipelas, or diffuse inflammation of the cellular membrane, or venous and arterial inflammation. Those sthenic inflammations, if I may use the expression, occur especially in those persons who have lost much blood. Let it be your object, therefore, in every operation, that it should be performed in such a manner that there should be as little waste of blood as possible."

To avoid unnecessary loss of blood in operations, beside a thorough knowledge of anatomy, Sir Benjamin advises, "when an operation is likely to be tedious, to take up the bleeding vessels as you go on; as, for example, in the dissection of some tumours, and even in some cases of amputation, when the patient has no blood to spare. Sometimes, where there is a long continued dissection, you will find great advantage from using a silver knife with as sharp an edge as can be given to this metal. The silver knife will divide the cellular membrane and smaller vessels, but it will not divide any vessel of considerable size. As it divides the cellular membrane it also stretches it, and elongates the vessels that are in it, and you know that vessels which are stretched before they are divided, bleed but little." The lecturer recommends that a tourniquet be always used in amputation; for although direct pressure, as with the thumb, may suffice to stop the bleeding from a large vessel, as the femoral artery, the tourniquet will in addition arrest the bleeding from the small vessels.

The present volume comprises thirty-nine lectures; six of which are devoted to the consideration of mortification; three are on diseases of the hip-joint; four on inflammation, ulceration, &c., of the veins; and the remainder to various subjects, as fistula in ano, hemorrhoids, and other diseases of the rectum; polypi; extraction of foreign bodies, etc. etc.

2 The work of Mr. Tamplin is also in the form of lectures. Originally, these lectures were delivered at the Royal Orthopœdic

Hospital in London, and published in the London Medical Gazette. Subsequently, "at the request of several friends," as the author informs us, he was induced to revise them, and republish them, with some additions, in a separate form. It is from this revised edition that we now have an American reprint, in a style which, bating the paper backs, (employed we suppose for the convenience of sending by post to subscribers) is quite agreeable to the eye. The illustrations are creditable specimens of wood engraving; the paper is white, with good wide margins to the pages; and the typography, but for a little carelessness in proof-reading, very fair.

The work consists of *fourteen lectures*, the first being confined to general introductory observations. Lecture 2 is on talipes equinus and its treatment; 3, on talipes varus congenitus and treatment; 4, talipes varus non-congenitus, talipes valgus, etc.; 5, talipes calcaneus, talipes calcaneo-valgus, and treatment; 6, 7, 8 and 9, treat of deformities of the knee and their treatment; 10, 11, 12 and 13, of rachitis and curvatures of the spine; 14, of wry neck, contraction of the lower jaw, shoulder-joint, arm, wrist, fingers, toes, etc. On all these subjects the work of Mr. Tamplin may be regarded as an excellent manual, embracing the latest improvements in apparatus and the best methods of cure.

3. The Elements of Surgery by Mr. Liston has been before the profession in this country several years, and extensively, too, as may be seen from the fact that the present is the *fourth* American edition, three of which, including this, under the supervision of Professor Gross. We can adduce no better evidence of its general approval. Mr. Liston is a bold and original thinker, with quite too good an estimate of himself to pay court to authority, however high, and too much regard for science and himself to do injustice to rivals and contemporaries. Well read, and enjoying extensive opportunities for acquiring experience, few living authors are better qualified for expounding the principles of surgical science, and the evidences of these attainments are abundant in the work before us.

The additions by Professor Gross are numerous, and always judicious—not impertinently thrust in, or inordinately eked out. The illustrations, although numerous and valuable, we regret to be obliged to say, are not all executed with that neatness for which our Philadelphia artists are distinguished.

4. The lectures on the Operations of Surgery by Mr. Liston, which are embraced in the present volume, were delivered in University College, London, in 1844, and published subsequently in the "London Lancet." Like the preceding work, it bears the impress of a master's hand. No man among British surgeons of the present day, probably, is more distinguished as an operator, than Robert Liston. His great dexterity, and apparent fondness for operating, has subjected him to the suspicion, by those not personally friendly to him, of less caution in resorting to the use of the knife, than the rules generally observed on that subject enjoin. With what justice we pretend not to know; but it has been boldly charged on him in some of the public prints in London, in canvassing his treatment of the case of the late Mr. Seaton, who was shot in a duel some months back, in which he tied up the external iliac artery. The patient having died shortly after the operation, the enemies of the surgeon have insisted that he was led astray in his diagnosis and treatment of the case, by what they allege to be his ruling passion. We are inclined to regard the charge as malicious, or designed to affect the legal issue before the courts, growing out of the breach of the law against homicide. Nevertheless, it shows the character enjoyed by Mr. L. as an extensive and expert operator. It is, however, a melancholy truth, that the love of operating, with those who follow it, "grows with their growth and strengthens with their strength," until arrived at that period when the value of all fictitious reputation comes to be duly estimated, and nothing remains to charm the mind but what is "lovely and of good report."

The love of display is natural to the human heart, and in that respect, operative surgery holds the same relation to other branches of the healing art that military duties do to civil offices. It represents "the pomp and circumstance" of our professional life. It is the tinsel, the glitter, that catches the eye and excites the huzza of the ignorant and unreflecting masses. Impelled by such influences, the ambitious surgeon is himself too apt to forget that "an operation is, for the most part, an abrupt and rude interference with nature"—an acknowledgment, in fact, of the imperfection of our art.

In the lectures before us we are glad to find that proper views on this subject are inculcated by Professor Mütter.

"To help the lame and to cure the blind, is but a small, though essential, part of the surgeon's duty, and is not to be accomplished by manual dexterity, by mere handiwork. You know full well that the surgeon of modern days has far higher duties to perform; that the art of healing is not divided, as in the darker ages, into the labour of the mind and the labour of the hands, the latter being our portion. The surgeon, it is true, was once the servant of the priest and the physician; he performed the menial part of dressing wounds, shaving the heads of his masters or their patients, and stooped to other meaner occupations. He shared neither the emoluments nor honours." This is the true doctrine. Teach surgeons to think they have offices of a higher grade to perform than mechanically lopping of a limb or tearing out a tumour, and they will look at, and teach their patients to regard, such exploits as the least intellectual and honorable of the functions they perform.

In this volume we have directions for the performance of most of the operations belonging to surgery, as well as instructions for the treatment of diseases and accidents requiring operations; the subjects of chief importance not embraced in the lectures of Mr. Liston being supplied by Dr. Mütter. The contributions of the latter occupy, indeed, nearly one-half of the pages of the volume, and contribute a full share to its interest.

Lecture I, which is by Dr. Mütter, is on the importance of operative surgery, and the necessity of a thorough knowledge of anatomy; incisions; hemorrhage; dressing wounds; union of wounds; sutures.

The subject of hemorrhages is treated at considerable length, and the various methods of arresting the flow of blood from all parts of the body are fully and clearly described, constituting, it seems to us, an excellent *resumé* of this branch of surgical practice. Affections of the eye, are likewise very fully treated of, partly by Mr. Liston, and partly by Dr. Mütter. We have been especially pleased with the pages devoted to *cataract*, in which Dr. Mütter seems to have collected, in the space of about thirty pages, every thing important that is known, as to its variety, pathology, and treatment. It is in fact the best summary with which we have any where met. But we cannot point out all the portions of the work which have struck us as particularly valuable, and must therefore be content with commending the volume, as well as that on the principles, to the particular notice of the profession, as comprising a great amount of solid information on the principles and practice of surgery.

---

*A Clinical Introduction to the Practice of Auscultation, and other modes of Physical Diagnosis; intended to simplify the study of the diseases of the Lungs and Heart.* By H. M. Hughes, M. D.; Fellow of the Royal College of Physicians; Assistant Physician to Guy's Hospital, etc. 12mo. pp. 270. Lea & Blanchard. 1846.

Dr. Hughes' work came from the London press during the past year, and is now the latest publication on the subjects of which it treats. Thus far, it has had the rare good fortune to be commended by all the critics who have spoken of it, which is pretty strong evidence that it possesses more than ordinary merit. No one can examine it without admitting that it is a very useful book, and well adapted to the wants of beginners in the study of the physical signs of disease. Less elaborate than the work of Barthe and Roger, it yet contains all that is essential as an introductory treatise, while it possesses an advantage over the excellent work by Dr. Walshe, which is confined to diseases of the lungs, in also embracing diseases of the heart.

"The means adopted for investigating diseases of the chest by physical signs, in contradistinction to the natural symptoms, are principally included under the terms Inspection, Palpation, Percussion, Auscultation, Mensuration, and Succussion." After a brief chapter containing "preliminary observations and directions," the author proceeds to treat of the subjects embraced in the volume in the order pointed out.

Dr. Hughes modestly disclaims either novelty or originality, but having read what has been written by those who have preceded him, and had the advantages derivable from fifteen years of practice and observation in one of the largest hospitals in London, he very justly claims the privilege of an independent observer, in stating the conclusions at which he has arrived.

---

*The Diagnosis, Pathology, and Treatment of the Diseases of the Chest.* By W. W. GERHARD, M. D.; Lecturer on Clinical Medicine to the University of Pennsylvania; one of the Physicians to the Pennsylvania Hospital, etc. 8vo. pp. 288. Second Edition, revised and enlarged. Ed. Barrington and Geo. D. Haswell. Philadelphia, 1846.

Although the present work of Dr. Gerhard is called a *second* edition, a considerable portion of it in fact appears before the public a third time. This has afforded to the author full opportunity for a careful revision; and that he has availed himself of this advantage is apparent in the style as well as the matter of his volume. Instead of being presented in the form of lectures, "in the present edition, the work is thrown into the usual form of chapters, divided according to the subjects of which they treat. The whole work has been revised, numerous corrections have been made, and a considerable quantity of new matter has been added," without materially increasing the size of the volume.

Dr. Gerhard's work differs much from that of Dr. Hughes. It is more comprehensive in its scope, more authoritative in its tone, and less doubtful in its conclusions. It is the production of one who has enjoyed extensive opportunities for studying the diseases it embraces, and hence presents strong claims to professional confidence.

